# Database Release: PPSDB, a Linked Open Data Knowledge Base for Protist–Prokaryote Symbioses

**DOI:** 10.1111/jeu.70049

**Published:** 2025-10-10

**Authors:** Brandon K. B. Seah

**Affiliations:** ^1^ Thünen Institute for Biodiversity Braunschweig Germany

**Keywords:** FAIR, knowledge graph, semantic web, SPARQL, Wikibase.cloud

## Abstract

As the ecological and evolutionary importance of symbiotic interactions between protists (microbial eukaryotes) and prokaryotes (bacteria and archaea) is better appreciated, keeping an overview of their diversity and the literature becomes a growing and ongoing challenge. Here I present the Protist‐Prokaryote Symbiosis Database (PPSDB), comprising 1146 manually curated interaction statements sourced from 443 publications, where biological taxonomy, anatomical localization, and analytical methods applied have been annotated and mapped to external databases and ontologies, such as Wikidata, NCBI Taxonomy, and Gene Ontology. I describe how its data model deals practically with challenges such as incomplete information and inconsistent taxon concepts, which will be applicable to similar projects. Both the model and underlying Wikibase software platform are highly extensible, so new items and properties can easily be added. Unlike a static table or list of citations, PPSDB is a structured knowledge base that enables programmatic access and powerful, integrated semantic queries. The database is available at https://ppsdb.wikibase.cloud/.

## 1 Introduction

The study of protists (microbial eukaryotes) has revealed a fascinating diversity of interactions with prokaryotes, including symbionts that defend their hosts, help them move, and orient them in the environment (Petroni et al. 2000; Hongoh et al. 2007; Monteil et al. 2019). New symbioses are regularly discovered, e.g., by mining protist genome data for prokaryote sequences (Davison et al. 2023), while previously described ones can be profitably revisited with modern methods. To contextualize new discoveries and spot larger trends and knowledge gaps, an accurate overview of the current body of knowledge is indispensable.

The existing entry points to knowledge on the diversity of protist‐prokaryote interactions are research articles and reviews, e.g., (Ball 1969; Bjorbækmo et al. 2020; Husnik et al. 2021; Kostygov et al. 2021; Shi et al. 2021; Fokin and Serra 2022). These are static, usually not interlinked with primary data, and formatted for human readers rather than programmatic queries, even if some information is presented in tabular form. It is therefore difficult to get a reliable answer to a question such as “what protists have Alphaproteobacteria symbionts localized in the host nucleus” without a deep dive into the literature for oneself.

The study of biotic interactions also suffers from poor discoverability of relevant information (Poelen et al. 2014; Mihara et al. 2016). Ideally, databases should be more than just a list of taxa or citations, but also capture other facets such as phylogenetic affiliation, interaction type, and environmental context. To achieve this, they should be built on a semantic data model that is extensible enough to accommodate new information, concepts, and terminology as they arise.

Specific challenges are posed by the ever‐evolving biological taxonomy and methods used to identify and describe organisms. Better taxon sampling and methods constantly drive updates to the higher taxonomy and nomenclature of both eukaryotes (Adl et al. 2019) and prokaryotes (Parks et al. 2020). For taxa originally identified or described on the basis of morphology or phenotype, their placement within modern taxonomies may be unclear, although some symbioses have been revisited with sequencing to clarify their phylogenetic identity (Boscaro et al. 2013; Schrallhammer et al. 2018). In contrast, many recent studies assign names solely from sequence data. Many organisms, particularly environmental microbes, never receive a formal scientific name and remain known under an informal or provisional name even if otherwise well characterized. Therefore, in addition to names and taxonomy, the analytical methods and evidence base behind each described symbiotic interaction should also be documented.

For the uses envisioned above, I argue that the information is best managed in the form of a knowledge graph. Knowledge graphs are data structures that represent concepts/entities and the relationships between them abstractly as the nodes and edges of a directed graph (network) (Chaudhri et al. 2022). A graph's structure is commonly specified as a collection of linkages, each comprising two nodes and the edge that connects them. The meanings assigned to nodes and edges depend on the domain‐specific application of the knowledge graph: for symbioses, nodes can represent biological taxa, and edges represent their interactions. Complex multi‐way or nested relationships as well as incomplete information can thus be represented more naturally and efficiently than in a tabular format or relational database.

Database items can be assigned standardized identifiers (uniform resource identifier, URI), and be further linked (“mapped”) to equivalent entities in other databases, e.g., of taxa or publications. A URI can be a web address that returns useful information about the entity. Data published following such principles are known as Linked Open Data (LOD) (Bauer and Kaltenböck 2012). Taxon names can be seen as analogous to URIs within biology, as they also aim to be unique and language‐agnostic identifiers for real‐world entities. Taxon names remain the primary vehicles through which biologists convey and retrieve information about organisms, albeit intended for human use and recall (Patterson et al. 2010). Machine‐readable URIs fulfill a similar role but can be processed programmatically. Linking equivalent concepts or entities between datasets with URIs lets us build on other databases and avoid duplicated effort; for example, we do not need to curate a full biological taxonomy in our own database if we map taxa to an existing, programmatically accessible taxonomic database. Other concepts/entities, such as anatomical and environmental terms, can be mapped to ontologies, such as the Gene Ontology (Gene Ontology Consortium et al. 2023) and Environment Ontology (Buttigieg et al. 2016). This not only ensures that terminology is used consistently with the wider community, but also allows sophisticated queries that take advantage of the semantic relationships encoded in those ontologies (Pacheco et al. 2022).

Here, I describe a knowledge base for protist‐prokaryote symbiotic interactions, and showcase how Linked Open Data principles enable powerful, integrated searches across multiple resources. The design objectives were to: (i) represent curated information from the scientific literature, with citations for each statement; (ii) focus on named symbiotic interaction partners from low‐diversity systems, rather than microbiome studies dealing with higher‐level taxa or OTUs; (iii) link records to sequence databases; (iv) enable multiple entry points for queries, including biological taxonomy, anatomical localization of symbionts, and analytical methods used to identify organisms; (v) map concepts and entities in the database to external taxonomies, ontologies, and identifiers, to ensure that they are described consistently and interoperable with other resources and knowledge representations.

## 2 Materials and Methods

### 2.1 Software Platform and Tools

The database was built on a Wikibase instance hosted by Wikibase.cloud, a service provided by Wikimedia Deutschland. The database was edited through the web interface, through batch edits using the QuickStatements tool (https://www.wikidata.org/wiki/Help:QuickStatements), and programmatically with Python scripts (https://github.com/kbseah/ppsdb‐utils/) using the WikibaseIntegrator library v0.12.5 (https://github.com/LeMyst/WikibaseIntegrator). The data dump to XML was performed with mediawiki‐dump‐generator (https://github.com/mediawiki‐client‐tools/mediawiki‐dump‐generator). Periodic exports for indexing by Global Biotic Interactions (GloBI) are hosted on GitHub (https://github.com/kbseah/ppsdb‐globi‐export). Network metrics for the symbiont co‐occurrence graph were calculated with networkx v3.4.2 (Hagberg et al. 2008).

### 2.2 Data Model and Terminology

**FIGURE 1:**
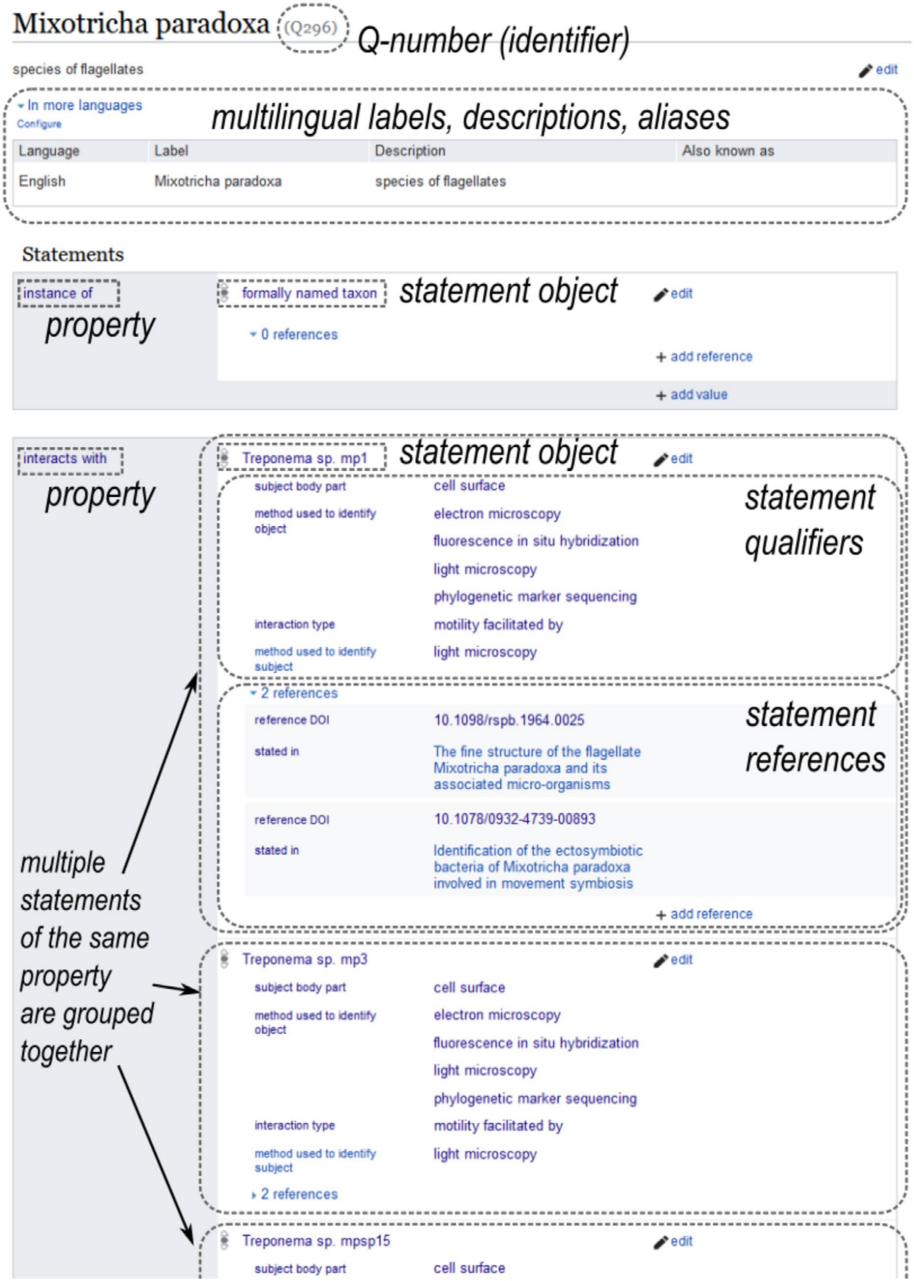
Annotated screenshot from the Wikibase item page for a host species, *Mixotricha paradoxa*, which illustrates how symbiotic interactions are represented as statements that link it to respective symbiont items, and which can be further qualified by additional information and references.

In the Wikibase platform, nodes are called “items”, and the edges connecting them are assigned specific meanings, or “properties”. Each connection (two items linked by a property) makes up a “statement”, with one item as the subject and the other as the object (Figure 1). Statements themselves can be treated like items and be the subject of further “qualifier” statements that provide additional information. Statements can also be annotated with references, which are a special type of qualifier statement.

Items may belong to one of two types, “classes” and “instances”, following the usage in Wikidata (https://www.wikidata.org/wiki/Help:Basic_membership_properties). A class is a set of items that have common properties; the members of a class are known as instances. Classes may be further subdivided into subclasses. For example, “ *Pelomyxa palustris* ” is an instance of the class “formally named taxon”, which is a subclass of “taxon”. Other classes in PPSDB represent references, organismal body parts, analytical methods, environmental terms, and interaction types; all items are ultimately descended from the root class “entity”.

**FIGURE 2:**
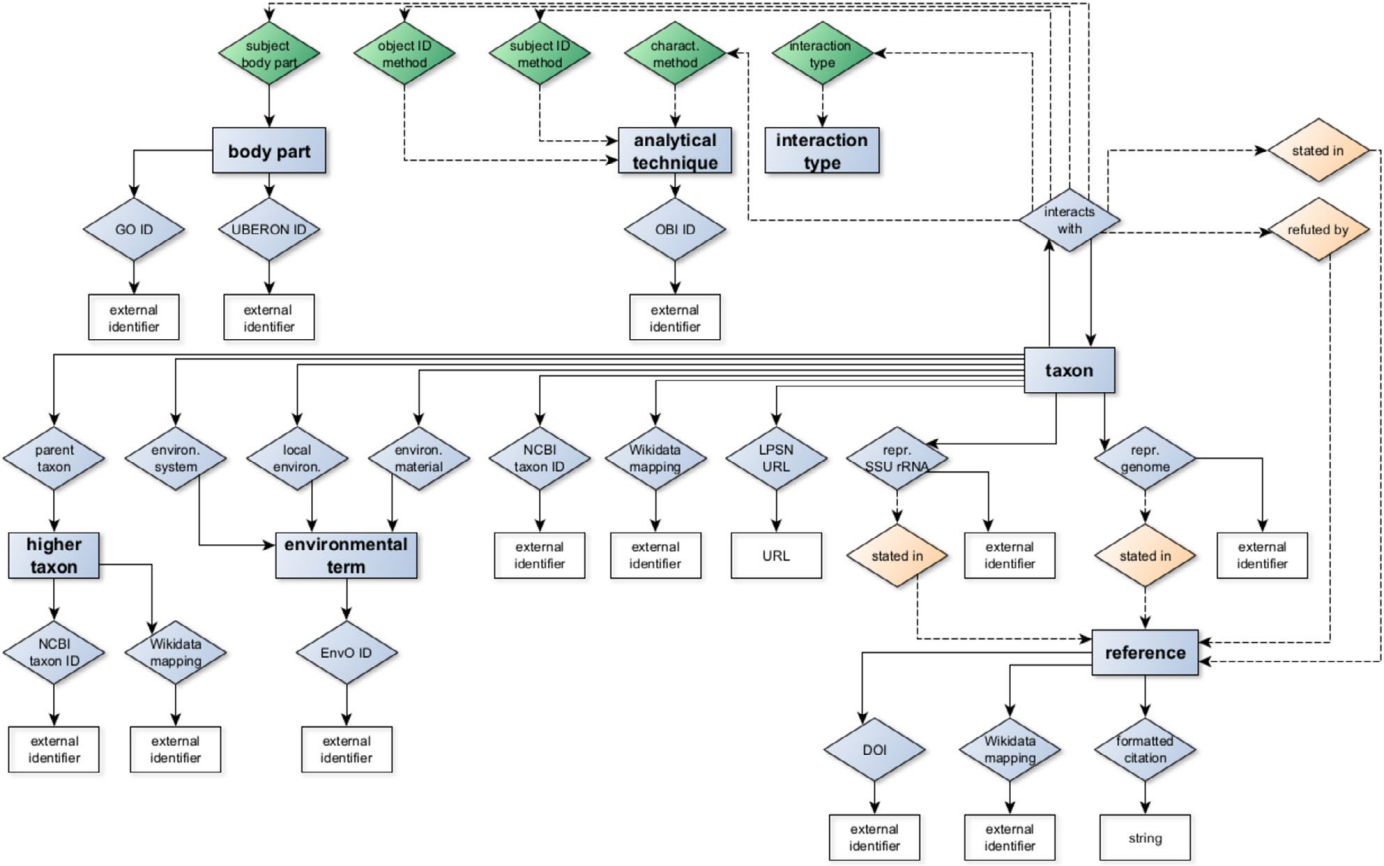
Relationships between items (blue rectangles), properties (diamonds), and other data types (white rectangles) in the PPSDB data model. Qualifier and reference relationships are depicted with dashed lines. Properties used in qualifiers or references are colored green and orange respectively. Subclasses of “taxon” and “environmental term” are not shown, for simplicity.

I modeled each biotic interaction as a statement linking two taxon items with an “interacts with” property (Figures 1 and 2). Each statement was further qualified with (i) where the symbiont is localized in the host organism/cell, (ii) the analytical methods used to identify (taxonomically and phylogenetically) host and symbiont, and (iii) the nature of the biotic interaction (e.g., transfer of fixed organic carbon, pathogenic), if known. Further statements on taxon items mapped them to external taxonomy databases and representative sequence records and described the environmental context from which organisms were isolated or sampled (Figures 1 and 2).

### 2.3 Data Collation and Mappings to External Identifiers

Reported symbiotic interactions between protists and prokaryotes were gathered from the published literature through ad hoc keyword searches and relevant review articles (Text S1). These included studies that specifically focused on symbiosis, as well as morphological or taxonomic studies that incidentally described associated microbes.

Relevant information was extracted from original research publications where possible, and mapped to external identifiers if a suitable exact match existed (Table 1). Taxon items were created to represent the interacting organisms; these are understood to be at species rank or below, even if they are only identified to a higher ranked taxon, similar to the concept of a “submittable taxon” in ENA (https://ena‐docs.readthedocs.io/en/latest/faq/taxonomy.html). This was to avoid potential conflation of multiple taxa under the same identifier. If sequence data were available, the taxon was linked to a representative sequence named in the publication describing it, to ensure that the name is associated with empirical data should disambiguation be necessary in the future. Equivalent identifiers in the NCBI Taxonomy (Schoch et al. 2020) were linked if available. Formally described or *Candidatus* (for prokaryotes) taxa were linked to Wikidata and the List of Prokaryotic Names with Standing in Nomenclature (LPSN) (Parte et al. 2020) on the basis of taxon name.

**TABLE 1:** Concepts or entities represented in the database and relevant external databases, ontologies, or identifiers that they are linked to, if exact matches are available.

Concept/entity	Relevant database, ontology, or identifier
Taxonomy of the interacting organisms	NCBI Taxonomy Wikidata List of Prokaryotic Names with Standing in Nomenclature (LPSN)
Localization of symbionts in the host organism	Gene Ontology UBERON
Nature of the biotic interaction, if known or inferred	OBO Relations Ontology
Analytical methods used to identify organisms or determine the interaction type	OBI Evidence Ontology
Environment where the host organism was collected or isolated	Environment Ontology
Publication describing the symbiosis	DOI Wikidata
Nucleotide sequence records	Genbank accession

The localization of symbionts in the host was mapped to cellular anatomy terms in the Gene Ontology (GO) (Ashburner et al. 2000; Gene Ontology Consortium et al. 2023) or metazoan anatomical terms in Uberon (for protists that are also symbionts of animals) (Mungall et al. 2012). The relationships between anatomical terms are represented in ontologies, which can be exploited when performing queries.

Cited publications were linked to digital object identifiers (DOIs) and to Wikidata, where bibliographic data are maintained by a community project, Wikicite. Citations missing from Wikidata are easily imported with the Scholia tool (Nielsen et al. 2017). Formatted citations were obtained from CrossRef from their DOIs; otherwise, they were added manually.

Three properties were used to describe the environmental context at different scales–broad scale environmental context, local environmental context, and environmental material–using Environment Ontology (EnvO) terms (Buttigieg et al. 2013, 2016), following the MIxS guidelines (https://github.com/EnvironmentOntology/envo/wiki/Using‐ENVO‐with‐MIxS) (Yilmaz et al. 2011).

Methods used to characterize the organisms were recorded so users can evaluate the evidence base supporting each statement. If equivalent terms existed, techniques were mapped to the Ontology for Biomedical Investigations (OBI) (Bandrowski et al. 2016), and interaction types were mapped to the OBO Relations Ontology (https://github.com/oborel/obo‐relations).

### 2.4 Challenges for Data Mapping and Modeling

Most challenges were related to taxonomy because informal or provisional names are often used for microorganisms, and disparate methods and levels of detail have been used to characterize them. Ideally, each organism would be described in a scientific publication under a formal taxon name, accompanied by published sequence accessions and an equivalent NCBI taxon item. A new Wikidata item for the taxon was created if it did not already exist. The PPSDB item was then linked to the corresponding Wikidata and NCBI Taxonomy items. Here I describe how I dealt with other cases that were not so neatly organized.

*Informal or provisional taxon names with NCBI Taxonomy equivalent*. Many studies described organisms without assigning a formal taxon name, but their phylogenetic affiliation is nonetheless known, sequence data are available, and the corresponding informally named taxon item in the NCBI Taxonomy appears to be equivalent to the taxon concept in the study. If so, the item was mapped to that NCBI taxon ID and labeled with the informal name used in the cited publication, as well as known aliases from other publications and databases.

*Taxon concept not in NCBI Taxonomy*, *but sequence data available*. Taxon items in the NCBI Taxonomy and taxonomic annotations of sequence records may not be up to date, or may differ from the published literature. For example, the ciliate species *Eufolliculina methanicola* (https://ppsdb.wikibase.cloud/entity/Q52) was formally described in a scientific publication (Pasulka et al. 2017), but sequences from that study were published in Genbank under a placeholder taxon “Folluculinidae sp.” (NCBI: txid1934002), which is used in the NCBI Taxonomy for records that were only identified to the family level and so may represent a mixture of different species. The PPSDB item was therefore not mapped to the NCBI Taxonomy, because it can lead to incorrect results if the identifier is used to retrieve sequence data. For this example, a formal taxon name was published, so a Wikidata item was created for it and mapped to PPSDB. To allow us to track the identity, should the NCBI Taxonomy be updated in the future, a representative SSU rRNA sequence record for this taxon that was cited in the original publication (KX012915) was linked to this item with the property “representative SSU rRNA sequence record”.

*Taxon name/concept with no sequence data available*. An organism may have been identified by morphology alone, without using sequencing methods, or sequence data produced in a study cannot be found. For example, a species of *Arcobacter* (https://ppsdb.wikibase.cloud/entity/Q410) was identified as a symbiont of *Bihospites bacati* (https://ppsdb.wikibase.cloud/entity/Q409), but although sequencing of a marker gene was reported, the sequence was not published. Alternatively, the organism may have been identified to a higher taxonomic group by its morphology or with methods such as group‐specific molecular probes. For example, the ciliate *Frontonia leucas* (https://ppsdb.wikibase.cloud/entity/Q1782) is associated with an unclassified Alphaproteobacteria (https://ppsdb.wikibase.cloud/entity/Q158) that was identified with group‐specific molecular probes. As no direct sequence data were available in these cases, there were no sequence records to anchor the taxon concept empirically (see above).

PPSDB items for such taxa were labeled with descriptive names based on what was reported in the publication, e.g., “unclassified Alphaproteobacteria”, but were not mapped to external identifiers. Similarly named *incertae sedis* items may exist in the NCBI Taxonomy (e.g., “unclassified Alphaproteobacteria”, NCBI: txid33807), but these were deliberately not mapped from PPSDB because they will pull in incorrect results if used for programmatic queries.

*Consistent mapping of items to higher taxonomy*. Not all organisms described in the literature could be mapped to external taxonomies, nor were they always identified to the species level. Nonetheless, I linked all taxon items to the next‐highest‐ranking, formally named parent taxon that was represented in both Wikidata and NCBI Taxonomy. This enabled consistent searches by taxonomy, even if the species themselves were not mapped to an external taxonomy. The parent taxon items are instances of a class “higher taxon” that are not used in interaction statements.

*Experimentally induced interactions*. A number of symbiotic microbes were first identified in one host species but maintained in the laboratory in a different host because the original host was not suitable for experiments. For example, *Acanthamoeba castellanii* has been used as a lab host for various intracellular bacteria isolated from other amoebae (Schulz et al. 2016). These experimentally induced interactions were represented with a different property, “interacts experimentally with”, to distinguish them from naturally occurring interactions.

## 3 Results and Discussion

PPSDB is hosted by Wikibase.cloud and is browsable through the web interface at https://ppsdb.wikibase.cloud/. A SPARQL endpoint is available for programmatic queries, with examples to help users get started: https://ppsdb.wikibase.cloud/query/. The structured data (in the Item: and Property: namespaces) is released under a CC0 1.0 public domain dedication (https://creativecommons.org/publicdomain/zero/1.0/).

### 3.1 Database Statistics

The database currently (27 September 2025) documents 1146 biotic interactions between 558 host items and 862 symbiont items, with 433 references cited. The number of citations is incidentally similar to the 328 works cited by Gordon H. Ball in his 1969 review, “Organisms living on and in Protozoa” (Ball 1969). However, given my focus on the phylogenetic identity of the symbiotic partners and linking them to sequence data, there is a bias towards more recent publications in PPSDB, and the overlap between the two sets of citations is minimal.

The most commonly represented host protist phyla are Ciliophora (158 taxon items), Metamonada (82), and Amoebozoa (54), while the most commonly represented symbiont prokaryotic phyla are *Pseudomonadota* (308 items), *Methanobacteriota* (75), and *Bacteroidota* (69). This undoubtedly reflects the activity of researchers rather than the abundance or ecological significance of these organisms. Some non‐protist, non‐prokaryote taxa are represented, e.g., termite hosts of metamonad flagellates that themselves have bacterial symbionts, as the host species helps to identify the flagellate. Multipartite interactions, or highly nested ones, are easily modeled, e.g., the bacterial epibionts of spirochaete ectosymbionts of flagellates from termite guts (Utami et al. 2019).

### 3.2 Database Queries With SPARQL

The Wikibase platform is bundled with a query engine, which uses the SPARQL language, which has similarities to the better‐known SQL. Whereas SQL is used with conventional relational databases, SPARQL is designed for knowledge graphs (Text S2). Examples: (1) Find instances of symbionts that are localized in the nuclei (or more specific compartments thereof) of their hosts (https://tinyurl.com/25fxfny9). (2) Find instances of symbionts that were identified through fluorescence in situ hybridization (FISH) but not sequencing of a phylogenetic marker gene (https://tinyurl.com/282s455g); FISH probes may give some information about taxonomic affiliation, but for microbes, a classification to species level is typically not possible without a marker sequence, so these symbioses may be worth revisiting. (3) Find symbiotic relationships described in publications co‐authored by a specific person (https://tinyurl.com/26a4kn2m); this “federated” query exploits mappings from PPSDB to Wikidata. Further examples are listed at https://ppsdb.wikibase.cloud/wiki/Project:SPARQL/examples.

### 3.3 Case Study: Exploring Co‐Occurrence Networks of Symbionts

In addition to organizing and retrieving information about symbiotic interactions, the flexibility of the data model and SPARQL queries allows users to gain new insights from the database.

**FIGURE 3:**
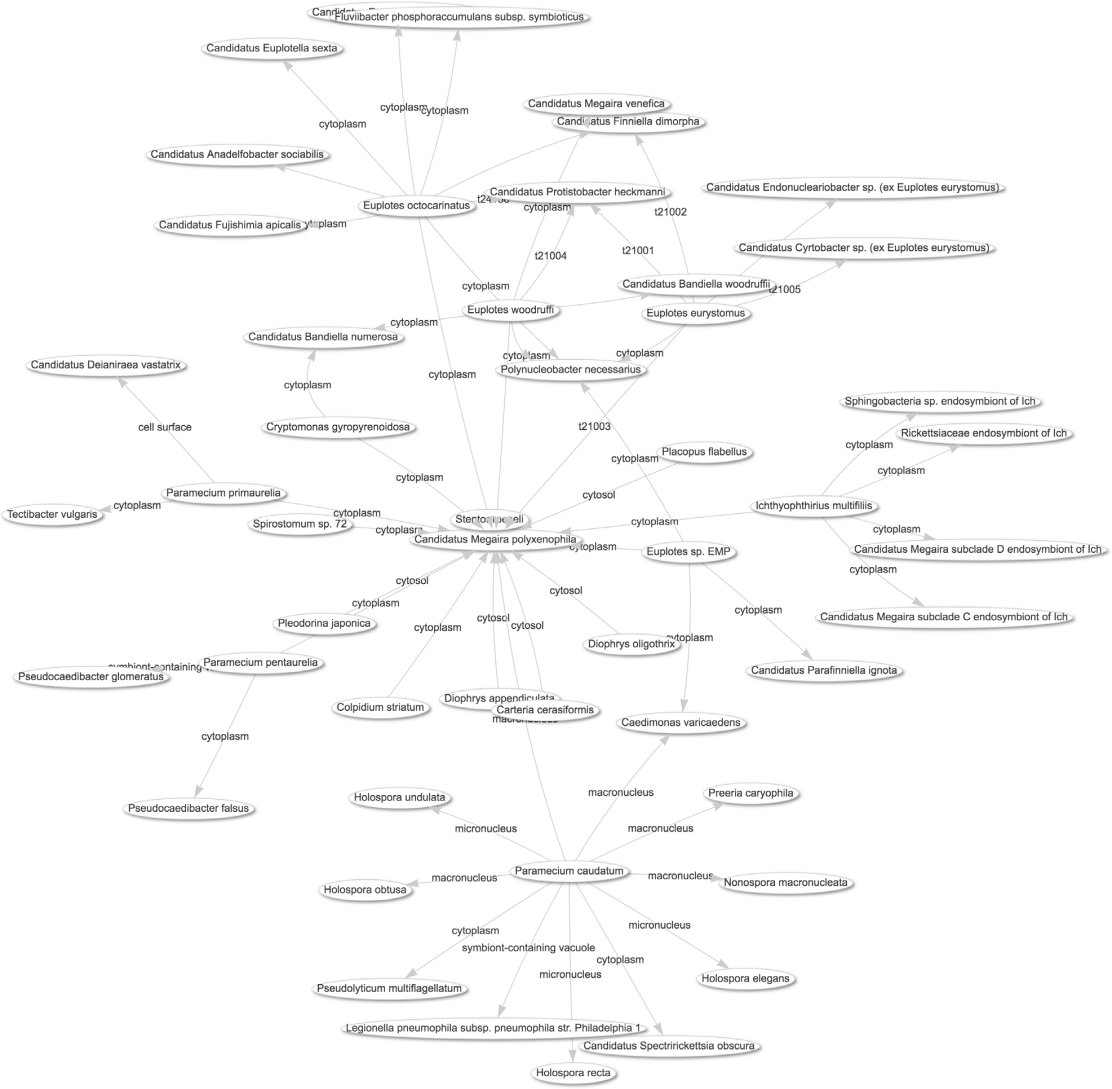
Graphical display of protist host species of the bacterial symbiont *Ca*. Megaira polyxenophila, and their other respective symbionts. The visualization was produced from a SPARQL query result by the Query Service engine included in Wikibase.cloud (https://tinyurl.com/2948dund).

Contrary to the popular perception of symbionts always being highly specific to a single host species, some microbial symbionts are found across multiple host species. A well‐known example from protist hosts is the alphaproteobacterium *Ca*. Megaira polyxenophila, which associates with diverse host eukaryotes including ciliates and green algae (Schrallhammer et al. 2013); many of these hosts also have other symbionts. These host‐symbiont connections linked to *Ca*. M. polyxenophila can be visualized as a network with a SPARQL query in PPSDB (https://tinyurl.com/2948dund, Figure 3), which shows that some of the symbionts, such as *Polynucleobacter necessarius* and *Ca*. Caedimonas varicaedens, are themselves also found in multiple hosts. This raises questions about host‐symbiont recognition, as well as the symbiotic niches occupied by these co‐occurring species. For example, do symbionts tend to co‐occur together in multiple hosts?

A simple way to identify the most “gregarious” symbionts is to count the other symbionts they co‐occur with. If symbionts are treated as nodes in a network, linked if they co‐occur in the same host species, this count corresponds to the degree of a node in network theory. Reported co‐occurrences of symbionts in the same host species were retrieved from the database (SPARQL query: https://tinyurl.com/24qfpozf). The network can be visualized in the query engine as an interactive graphic; clicking on a node opens the database page for that item. For further analyses with third‐party tools, query results can be exported to a table format; here, other network metrics based on the co‐occurrence graph (Table S1) were calculated with networkx (Hagberg et al. 2008). I focus on the 13 symbionts reported from more than one host species, though those from only one host are included when calculating network metrics.

As expected from the previous visualization, the two symbionts with the most co‐occurrences were *Ca*. M. polyxenophila (node degree 27) and *P. necessarius* (degree 20). Other metrics can give us different insights into the data. The PageRank metric, originally developed to rank web search engine results, measures centrality, i.e., how much of the network is linked through a given node. The local clustering coefficient measures the density of links between neighboring nodes; for this co‐occurrence network, if a group of species tends to co‐occur with each other, their nodes will be closer to forming a clique and have a higher local clustering coefficient. Ranking by these metrics throws up some species whose connection patterns are interesting even though they may have fewer connections overall (lower degree).

*Ca*. Armantifilum devescovinae, a symbiont of termite gut flagellates, has the highest PageRank (0.0173) although its degree is only 7. Strikingly, its clustering coefficient is zero. This is because none of the species it co‐occurs with also co‐occur with each other, but only with *Ca*. A. devescovinae. Such a pattern may suggest mutual exclusion, e.g., if its co‐symbionts have overlapping niches, or could indicate that *Ca*. A. devescovinae actually comprises multiple species, instead of different strains as originally reported (Desai et al. 2010). *Ca*. Endomicrobium trichonymphae, another termite gut flagellate symbiont, also has an unexpectedly high PageRank (0.00739) for its degree (5), but a nonzero clustering coefficient (0.40). This is because two host species reportedly contain *Ca*. E. trichonymphae alongside other symbionts, but unlike *Ca*. A. devescovinae, each has more than one co‐symbiont.

These metrics should not be taken at face value because the information is aggregated from heterogeneous sources (see “Challenges for data mapping and modeling”). Users can evaluate the evidence base behind reported symbioses with information collated in PPSDB. For example, from its PPSDB entry and the link to NCBI Taxonomy, a user can see that *Ca*. A. devescovinae has been described from marker sequences but not whole genome sequences that could address the question of its genetic variability. Moving forward, metagenomics‐based screening studies contribute an increasing number of new symbiont reports, so it will be important to distinguish such reports from those with morphological evidence. Furthermore, species concepts (or OTU delimitation) can significantly impact our interpretations. If *Ca*. Megaira polyxenophila from different hosts, usually considered different strains of one species (Lanzoni et al. 2019), were instead considered a species complex, the connectivity would change substantially. Species concepts of hosts may also change, e.g., the *Paramecium aurelia* complex, historically important for ciliate genetics and symbiosis.

### 3.4 Choice of Software Platform

I chose Wikibase as the platform for this database because it has both a web browser‐based interactive interface and an API for programmatic access, and is available as a cloud service. Wikibase was originally developed as the backend for Wikidata, the largest open knowledge graph. As such, its design caters to the Wikidata model, but this potential limitation was outweighed by its ease of use, active user community, ongoing support and development, and integration of a SPARQL engine and other tools. Existing tools and libraries to work with Wikibase can be applied instead of reinventing the wheel. The ease of federated searches with Wikidata was also an advantage. Many current Wikibase users come from the cultural heritage field (Diefenbach et al. 2021; Huaman et al. 2023; Shimizu et al. 2023), and include institutions like the European Union and the German National Library. PPSDB shows that an application in the natural sciences is straightforward.

Technical requirements and know‐how remain a hurdle to the adoption of knowledge graphs. Wikibase.cloud is a good compromise for smaller projects and prototypes driven by subject‐matter experts who may not have a deep background in semantic web technologies. No programming experience is required to get started, as data entry and editing can be performed through the web interface, with users learning additional tools (e.g., QuickStatements for tabular data entry, SPARQL for queries) as they go along. User management, project planning, and discussion pages can be maintained on the same wiki as the database itself, making it self‐contained. Other projects with similar aims have used tabular file formats, e.g., PIDA (Bjorbækmo et al. 2020), or built bespoke software, e.g., AQUASYMBIO http://www.aquasymbio.fr/, Viral Host Range DB https://viralhostrangedb.pasteur.cloud/ (Lamy‐Besnier et al. 2021), and Virus‐Host DB https://www.genome.jp/virushostdb/ (Mihara et al. 2016). Such software is harder to maintain in the long term and requires more effort to integrate with other linked data sets. Similar considerations have been cited by Wikibase users who have migrated from other platforms (Koho et al. 2023).

### 3.5 Virtuous Cycles of Data Curation

During data curation, I found outdated records or errors while mapping items to NCBI Taxonomy and Wikidata. I edited Wikidata directly, while the NCBI Taxonomy team was contacted by email with corrections. Commonly encountered issues included NCBI Taxonomy records that still used a provisional name although a formal taxon name or Candidatus name has been published, and taxon names or publications that were not yet represented on Wikidata. Linked open data naturally fosters collaboration and a mutualistic relationship between the linked resources, such that the curation and data cleaning of one benefit the others too (Seah 2023; von Mering et al. 2023).

### 3.6 Data Sharing and Archiving

Even if data are linked and open, interested users may not be able to find them easily. The core interaction data in PPSDB were therefore exported as a table for indexing by the Global Biotic Interactions (GloBI) database (https://www.globalbioticinteractions.org/), an aggregator for species interaction data that is searchable from its website and through an R package (Poelen et al. 2014). This increases the visibility of protists, which are underrepresented in ecological studies, and of the original publications, which are cited in full. To secure the long‐term availability of the database, periodic XML dumps are archived on Internet Archive. The export for GloBI is also archived separately on Zenodo.

### 3.7 Remaining Challenges for Data Modeling/Mapping

Some types of statements in the literature remain difficult to represent formally in my data model. Broad statements about higher taxa, e.g., “all *Kentrophoros* species are associated with bacteria from genus *Candidatus* Kentron”, are represented in the data model by creating individual items and links for each known species within those taxa. Such a statement implies that as‐yet unstudied or undescribed host species will also be found to interact with corresponding symbiont species. However, one cannot create items for unknown species, so I conservatively did not add such implicit statements.

The modeling of facets other than biological taxonomy is relatively basic and can be further developed. For symbiont localization, I currently do not distinguish between different types of topological relationships. For example, methanogenic endosymbionts are typically located in the host cytoplasm close to hydrogenosomes, but this detail is not captured by the single “subject body part” property. For interaction types, most terms have not been mapped to the OBO Relations Ontology (RO), because many terms that are meaningful to microbial ecologists, e.g., “syntrophy” and “auxotrophy”, do not appear in RO. The outcome (e.g., mutualistic vs. parasitic) and function of many microbial interactions are also unclear or only inferred. Nonetheless, more elaborate modeling of these aspects may be overly complex for most users, so a simpler representation may be more useful.

Microbiome survey studies were excluded from the scope of PPSDB. However, some larger protists are associated with diverse prokaryotes; some may be stable partnerships while others are facultative. Sequencing surveys may reveal dozens of such interactions per host species—should they all be included in the database?

Finally, there are the practical hurdles in extracting relevant information from publications. Taxon names and sequence accessions may be scattered throughout a manuscript and Data S1 or even across multiple publications; different names may be used for the same organism, methods may be incompletely reported, and in a few (thankfully rare) cases, which symbiont belongs to which host was not reported at all even though both are separately characterized. I suggest that authors summarize biotic interaction results in tabular format where any sequence accessions or identifiers are also directly listed.

### 3.8 Future Directions

Beyond the database itself, I aimed to demonstrate with PPSDB how knowledge graphs are especially well‐suited to modeling biotic interactions and integrating biological data, and to describe how I addressed specific challenges such as taxonomic inconsistency, uncertainty, and the proliferation of names and identifiers. I invite colleagues to contribute to PPSDB by editing the database or by alerting me to symbiotic interactions not represented within, and to consider adopting knowledge graphs for their own projects.

The model can easily be adapted to other types and facets of biotic interactions by adding new classes and properties. For example, a statement representing an allelopathic interaction between two plant species could have a qualifier that links to the phytochemical responsible, the latter represented as an item of class “chemical” and mapped to databases like ChEBI. PPSDB itself can be extended to encompass other taxonomic groups; viruses are particularly relevant as some giant viruses of protists were initially thought to be bacterial symbionts (la Scola et al. 2003). As mentioned above, a limited number of non‐protist, non‐prokaryote taxa are already represented in the database.

Future workers may choose to model interactions differently, or set up new knowledge graph projects on related topics like those mentioned above. To keep the work usable beyond the conclusion of a project, I recommend that new knowledge graph projects integrate themselves with the wider ecosystem from the start by following the Linked Open Data paradigm, i.e., mapping entities between databases, especially Wikidata, and also that they keep data findable and accessible in the long term through integration with GloBI.

The current bottleneck in data curation is in discovering and parsing the relevant scientific literature. Other projects have data‐mined molecular sequence metadata, e.g., the “host” tag in Genbank records, to produce large‐scale species interaction datasets (Wardeh et al. 2015; Albrycht et al. 2022). Such pipelines are most suitable for taxa, such as viruses, that are routinely described with sequencing, where relevant metadata are generally accessible in standardized form. We are however also interested in the historical literature, and in symbioses described with other methods, such as microscopy, where data deposition and metadata reporting are not yet as standardized as for sequencing. For the foreseeable future, we still need humans to verify that the data are reliable, but this could be supplemented by provisional interaction claims derived from data mining. Natural language processing could also help screen for relevant publications in full text databases such as Europe PMC.

## Supporting information


**Text S1:** Key review articles and search keywords.
**Text S2:** Introduction to SPARQL queries on Wikibase.

**Table S1:** Network metrics for symbionts reported from two or more host species, for the network of symbiont co‐occurrences in PPSDB.

## Data Availability

Main URL for PPSDB: https://ppsdb.wikibase.cloud/. Export to tabular format for indexing in GloBI: https://github.com/kbseah/ppsdb‐globi‐export, archived on Zenodo: https://doi.org/10.5281/zenodo.12687626. Scripts for database maintenance: https://github.com/kbseah/ppsdb‐utils, archived on Zenodo: https://doi.org/10.5281/zenodo.12805883. XML export of the entire PPSDB database on Internet Archive (16 Aug 2024): https://archive.org/details/wiki‐ppsdbwikibasecloud_w.
