# Supplementary material for: Database Release: PPSDB, a Linked Open Data Knowledge Base for Protist–Prokaryote Symbioses
Source: J Eukaryot Microbiol. 2025 Oct 10;72(6):e70049. doi: 10.1111/jeu.70049 (PMC12512223; doi:10.1111/jeu.70049)
Supplement: Supplementary file 1 — Text S1: Key review articles and search keywords. Text S2: Introduction to SPARQL queries on Wikibase. [file JEU-72-e70049-s001.pdf]

Text S1 Key Review Articles and Search Keywords

Review Articles

Amin S. A., Parker M. S. & Armbrust E. V. 2012. Interactions between diatoms and bacteria. Microbiol. Mol. Biol. Rev. 76:667–684.

Anderson O. R. 2012. Living together in the plankton: a survey of marine protist symbioses. Acta Protozool. 52:1–10.

Ball G. H. 1969. Organisms living on and in protozoa. In: Research in Protozoology. Elsevier. p. 565–718.

Bjorbækmo M. F. M., Evenstad A., Røsæg L. L., Krabberød A. K. & Logares R. 2020. The planktonic protist interactome: where do we stand after a century of research? ISME J., 14:544–559.

Brune A. & Dietrich C. 2015. The gut microbiota of termites: digesting the diversity in the light of ecology and evolution. Annu. Rev. Microbiol. 69:150720190645000.

Decelle J., Colin S. & Foster R. A. 2015. "Photosymbiosis in marine planktonic protists," *In*: Ohtsuka S., Suzaki T., Horiguchi T., Suzuki N. & Not F. (eds.) Marine Protists. Tokyo: Springer Japan. p. 465–500.

Dziallas C., Allgaier M., Monaghan M. T. & Grossart H.-P. 2012. Act together—implications of symbioses in aquatic ciliates. Front. Microbiol. 3:288.

Fokin S. I. 2004. Bacterial endocytobionts of Ciliophora and their interactions with the host cell. Int. Rev. Cytol. 236:181–249.

Fokin S. I. 2012. Frequency and biodiversity of symbionts in representatives of the main classes of Ciliophora. Eur. J. Protistol. 48:138–148.

Fokin S. I. & Görtz H.-D. 2009. "Diversity of Holospora bacteria in Paramecium and their characterization." *In*: Fujishima M. (ed.) Endosymbionts in Paramecium. Berlin, Heidelberg: Springer Berlin Heidelberg. p. 161–199.

Fokin S. I. & Serra V. 2022. Bacterial symbiosis in ciliates (Alveolata, Ciliophora): Roads traveled and those still to be taken. J. Eukaryot. Microbiol., 69:e12886.

Gast R. J., Sanders R. W., & Caron D. A. 2009. Ecological strategies of protists and their symbiotic relationships with prokaryotic microbes. Trends Microbiol. 17:563–569.

Görtz H.-D. & Brigge T. 1998. Intracellular bacteria in protozoa. Naturwissenschaften 85:359–368.

Görtz H.-D. & Fokin S. I. 2009. "Diversity of endosymbiotic bacteria in Paramecium," *In*: Fujishima M. (ed.) Endosymbionts in Paramecium. Berlin, Heidelberg: Springer Berlin Heidelberg. p. 131–160.

Husnik F., Tashyreva D., Boscaro V., George E. E., Lukeš J. & Keeling P. J. 2021. Bacterial and archaeal symbioses with protists. Curr. Biol., 31:R862–R877.

Jeon K. W. 2006. “Prokaryotic symbionts of amoebae and flagellates,” *In*: Dworkin M., Falkow S., Rosenberg E., Schleifer K.-H. & Stackebrandt E. (eds.) The Prokaryotes. New York, NY: Springer New York. p. 1028–1037.

Kostygov A. Y., Karnkowska A., Votýpka J., Tashyreva D., Maciszewski K., Yurchenko V. & Lukeš J. 2021. Euglenozoa: taxonomy, diversity and ecology, symbioses and viruses. Open Biol., 11:200407.

Nowack E. C. M. & Melkonian M. 2010. Endosymbiotic associations within protists. Philos. Trans. R. Soc. B Biol. Sci. 365:699–712.

Ossipov D. V., Karpov S. A., Smirnov A. V. & Rautian M. S. 1997. Peculiarities of the symbiotic systems of protists with diverse patterns of cellular organisation. Acta Protozool. 36:3–21.

Radek R. 2010. “Adhesion of bacteria to protists,” *In*: König H., Claus H. & Varma A. (eds.) Prokaryotic Cell Wall Compounds. Berlin, Heidelberg: Springer Berlin Heidelberg. p. 429–456.

Schulz F. & Horn M. 2015. Intranuclear bacteria: inside the cellular control center of eukaryotes. Trends Cell Biol. 25:339–346.

Schweikert M., Fujishima M. & Görtz, H.-D. 2013. “Symbiotic associations between ciliates and prokaryotes,” *In*: Rosenberg E., DeLong E. F., Lory S., Stackebrandt E. & Thompson F. (eds.) The Prokaryotes. Berlin, Heidelberg: Springer Berlin Heidelberg. p. 427–463.

Shi Y., Queller D. C., Tian Y., Zhang S., Yan Q., He Zhili, He Zhenzhen, Wu C., Wang C. & Shu L. 2021. The Ecology and Evolution of Amoeba-Bacterium Interactions. Appl. Environ. Microbiol., 87:e01866-20.

Search Terms

PubMed was searched for “protist AND symbiosis” and “[taxon name] AND symbiosis” where “[taxon name]” were class- and phylum-level taxa listed in Adl et al., 2019.

Specialist journals in protistology and microbiology were searched with the term “symbiosis”: *Journal of Eukaryotic Microbiology*, *European Journal of Protistology*, *Protist*.

Text S2 Introduction to SPARQL Queries on Wikibase

SPARQL (SPARQL Protocol and RDF Query Language; a recursive acronym) is a query language for knowledge graphs, specifically those that follow a standard known as the Resource Description Framework (RDF).

Some elements of SPARQL, especially its syntax and use of keywords like SELECT, WHERE, and GROUP BY, resemble the Structured Query Language (SQL) used with conventional relational databases. However, unlike relational databases, which are built on rectangular tables, knowledge graphs are built on statements, each of which comprises a "triple" of subject-property-object.

Let's start with basic SPARQL examples using Wikidata, before going through some PPSDB queries in more detail.

Further resources:

- Wikidata SPARQL tutorial https://www.wikidata.org/wiki/Wikidata:SPARQL_tutorial
- SPARQL Wikibook https://en.wikibooks.org/wiki/SPARQL

Basic example

Consider a knowledge graph that contains the following (pseudocode) statements, structured as triples of "subject–property–object":

```
basketball instance_of type_of_sport.

tennis instance_of type_of_sport.

basketball uses basketball_ball.

basketball max_players 10.

tennis uses tennis_ball.

tennis uses tennis_racket.

tennis max_players 4.
```

Entities in a knowledge graph can be broadly classified as being either items (e.g. basketball, tennis_ball; think "noun") or properties (e.g. instance_of, max_players; think "verb"). Each triple is therefore like a simple sentence in English: subject-verb-object.

The following query looks for all instances of sports that are played with a racket:

```
SELECT ?item WHERE {

  ?item instance_of type_of_sport.

  ?item uses tennis_racket.

}
```

"SELECT" and "WHERE" are keywords that are part of the SPARQL language, and are usually written in uppercase.

Most queries start with the keyword "SELECT" and a list of variables.

In this query, the only variable is ?item. Variables represent the entities that we wish to retrieve with our query and are prefixed with a question mark.

The keyword "WHERE" is followed by the criteria used to find the variables, grouped together between the curly braces "{}". The criteria in this query take the form of triple statements.

Translated to English, the query reads: "Select all items in the database, where the item is an instance of a sport, and it is played with a racket."

URIs and prefixes

In an actual database following the RDF framework, the items and properties are not plain English words like "basketball" and "sport" but instead represented by a Uniform Resource Identifier (URI). These look like web addresses (URLs) though they don't always resolve to a valid webpage. For example, in the Wikidata knowledge graph, which is a free and open database that is not limited to any specific knowledge domain, the concept of "basketball" is represented by an item with the URI: http://www.wikidata.org/entity/Q5372.

The statement "basketball instance_of type_of_sport" in the Wikidata database is represented as the following triple of URIs, corresponding to their respective concepts:

```
<http://www.wikidata.org/entity/Q5372> <http://www.wikidata.org/prop/direct/P31>
<http://www.wikidata.org/entity/Q31629>.
```

Note that:

- They all start with the same "http://www.wikidata.org/" domain
- Items are prefixed with "http://www.wikidata.org/entity/" followed by an identifier starting with Q.
- Properties are prefixed with "http://www.wikidata.org/prop/direct/" followed by an identifier starting with P.
- The statement above uses the Turtle (https://www.w3.org/TR/turtle/) syntax, where URIs are enclosed in angle brackets.

One advantage of using URIs instead of plain words to name items and properties is that URIs are (or should be) language-independent, as a given URI can have labels in multiple languages. It also avoids confusion when different entities have the same name but refer to different concepts, e.g. the game "basketball" (http://www.wikidata.org/entity/Q847) vs. the ball itself (http://www.wikidata.org/entity/Q810345).

Using URIs, the SPARQL query in the previous section can be rewritten:

```
SELECT ?item WHERE {

  ?item <http://www.wikidata.org/prop/direct/P31> <http://www.wikidata.org/entity/Q31629>.

  ?item <http://www.wikidata.org/prop/direct/P2283> <http://www.wikidata.org/entity/Q153362>.

}
```

Try the above query on Wikidata: https://w.wiki/Dxqb

To save on typing and improve readability, abbreviations for URI prefixes can be defined at the head of a query and the short forms used thereafter:

```
PREFIX wd: <http://www.wikidata.org/entity/>

PREFIX wdt: <http://www.wikidata.org/prop/direct/>

SELECT ?item WHERE {

  ?item wdt:P31 wd:Q31629.

  ?item wdt:P2283 wd:Q153362.

}
```

The prefixes in this example are for entities in the Wikidata database; wd: and wdt: are prefixes used for items and properties respectively.

Filter expressions

Statements can take strings, numerical values, and other parameters as predicates, not just items, for example the Wikidata property wdt:P1873 "maximum number of players", takes a numerical value. Such queries can be constructed by first retrieving the relevant statements with a variable, here ?max_players, then using the FILTER keyword to apply an inequality or Boolean expression.

```
PREFIX wd: <http://www.wikidata.org/entity/>

PREFIX wdt: <http://www.wikidata.org/prop/direct/>

SELECT ?item WHERE {

  ?item wdt:P31 wd:Q31629. # instances of "type of sport"

  ?item wdt:P1873 ?max_players. # with statement on the property "maximum number of players"

  FILTER ( ?max_players < 10 ). # where the value is less than ten

}
```

Example SPARQL queries in PPSDB

For more information on SPARQL queries beyond the basics outlined above, refer to the Wikidata SPARQL tutorial: https://www.wikidata.org/wiki/Wikidata:SPARQL_tutorial. PPSDB is built on Wikibase, the same software platform used by Wikidata, so the architecture and query syntax are very similar.

List all symbiotic hosts and their environment terms

PPSDB attempts to describe the environment where a symbiotic organism is found with properties "environmental material", "local environmental context", and "environmental system".

Let's find symbiotic hosts and any statements about their environment if available.

```
PREFIX pp: <https://ppsdb.wikibase.cloud/entity/>

PREFIX ppt: <https://ppsdb.wikibase.cloud/prop/direct/>
```

```
SELECT DISTINCT ?item ?itemLabel ?envmat ?envmatLabel ?envloc ?envlocLabel ?envsys ?envsysLabel WHERE
{

# Find any item that “interacts with” (property P19) another item, i.e. is a symbiotic host.

# In PPSDB, we use the convention that the subject of an “interacts with” statement is the “host”, while the object is the “symbiont”.

  ?item ppt:P19 ?symbiont.

# Use the OPTIONAL keyword because not all items have statements about their environment

  OPTIONAL { ?item ppt:P36 ?envmat }

  OPTIONAL { ?item ppt:P40 ?envloc }

  OPTIONAL { ?item ppt:P38 ?envsys }

# Display the item label in default language or English.

# Without this line, only the identifiers will be shown, which are not very readable for humans.

  SERVICE wikibase:label { bd:serviceParam wikibase:language "[AUTO_LANGUAGE],en". }

# Sort the output (alphabetically) by the item label.

} ORDER BY ?itemLabel
```

Find symbiotic interactions and their localization in the host cell (using qualifiers)

Statements are limited to a triplet of subject-property-object, such as “species_A interacts_with species_B”. What if there are some additional details that cannot fit into a simple triplet? In Wikibase, statements can be extended with additional information through qualifiers, which make the whole statement the subject of another statement. For example, the above statement can be qualified as “(species_A interacts_with species_B) subject_body_part cytoplasm” to represent the fact that this interaction takes place in the host cytoplasm.

In PPSDB, the most important property is P19 “interacts with”, which links host and symbiont items. “interacts with” statements can be qualified with information about the localization of the symbiont within the host (P20 “subject body part”) and the methods used to characterize adn identify the symbiotic partners (P22 “method used to identify object”, and P42 “method used to identify subject”).

```
PREFIX pp: <https://ppsdb.wikibase.cloud/entity/>

PREFIX ppt: <https://ppsdb.wikibase.cloud/prop/direct/>

PREFIX pps: <https://ppsdb.wikibase.cloud/prop/>

PREFIX ppss: <https://ppsdb.wikibase.cloud/prop/statement/>

PREFIX ppsq: <https://ppsdb.wikibase.cloud/prop/qualifier/>

SELECT DISTINCT ?localization ?localizationLabel (COUNT (DISTINCT ?interaction) AS ?count)  WHERE {

# Instead of pointing directly to the object as in previous queries, we retrieve the whole statement with property P19 “interacts with” as ?interaction.

  ?host pps:P19 ?interaction.
```

```
# Get the object of the statement as ?symbiont

  ?interaction ppss:P19 ?symbiont.

# Get any qualifiers under property P20 “subject body part” as ?localization

  ?interaction ppsq:P20 ?localization.

  FILTER (!ISBLANK (?localization))

  SERVICE wikibase:label { bd:serviceParam wikibase:language "[AUTO_LANGUAGE],en". }

} GROUP BY ?localization ?localizationLabel
```

Find members of a higher taxon (federated search with Wikidata)

PPSDB was designed to model information about symbiotic relationships, with the organisms identified to species level where possible. It was impractical to include in PPSDB a complete taxonomy of all the featured species. Instead, I chose to cross-reference species and their immediate higher taxa to Wikidata, which does have information about biological taxonomy. Therefore, to search PPSDB by taxonomy, one has to split the query into two parts: a subquery of Wikidata to retrieve relevant taxon items, and a subquery of PPSDB to retrieve taxon items that are mapped (cross-referenced) from Wikidata and with other query parameters.

The following query lists symbiotic interactions where the host organism belongs to the taxon Blattodea (cockroaches and termites). We first retrieve from Wikidata all lower taxa contained in Blattodea, then find PPSDB items that are mapped to them and also linked to items representing symbiotic hosts.

```
PREFIX pp: <https://ppsdb.wikibase.cloud/entity/>

PREFIX ppt: <https://ppsdb.wikibase.cloud/prop/direct/>

PREFIX wd: <http://www.wikidata.org/entity/>

PREFIX wdt: <http://www.wikidata.org/prop/direct/>

SELECT DISTINCT ?host ?hostLabel ?symb ?symbLabel ?wditem

# The WITH clause first runs a subquery of the Wikidata database; note Wikidata prefixes wd: and wdt:.

# The results of this subquery, contained in a list %wd of items potentially cross-referenced in PPSDB, are then passed to the subquery on PPSDB.

WITH {

  SELECT DISTINCT ?wditem WHERE {

# SERVICE specifies the address to Wikidata’s SPARQL engine.

    SERVICE <https://query.wikidata.org/sparql> {

# Search for items with parent taxon (P171) is Blattodea (Q25309).

# The asterisk means that we also want to chain together zero or more “parent taxon” relationships: the item Blattodea itself, items with parent taxon Blattodea, items whose parent taxon is an item whose parent taxon is Blattodea, etc.

      ?wditem wdt:P171* wd:Q25309.
```

```
    }
  }
} AS %wd
# The part of the query within the WHERE clause now searches PPSDB, using items cross-referenced from the Wikidata sub-query above.
WHERE {
  INCLUDE %wd
# Find PPSDB items representing host species (?host) whose parent taxon (?parent) is cross-referenced to one of the shortlisted taxa in Wikidata from the previous subquery (?wditem).
  ?host ppt:P19 ?symb.
  ?host ppt:P29 ?parent.
  ?parent ppt:P2 ?wditem.
  ?host rdfs:label ?hostLabel.
  ?symb rdfs:label ?symbLabel.
} ORDER BY ?symbLabel ?hostLabel
```

Example queries from main article

Here I will explain the example queries listed in the main article in more detail.

Find symbionts localized in host nuclei, or more specific compartments in nucleus

This search showcases how a query can use semantic content in the database and integrate an external database in the search.

The localization of intranuclear symbionts may be reported simply as “nucleus”, or more specifically as “nuclear envelope lumen”, or “macronucleus” (in ciliates, which have two developmentally distinct types of nuclei). The relationships between these terms are modeled in the database, e.g. nuclear envelope lumen is a part of, and macronucleus is a subclass of “nucleus”. Users can be more or less specific as required.

We have chosen not to maintain a full biological taxonomy within PPSDB, but instead to map taxa to Wikidata and the NCBI Taxonomy (see “Challenges for data mapping and modeling” above). The search is therefore executed as a federated SPARQL query across both PPSDB and Wikidata. Most described intranuclear symbionts are Alphaproteobacteria from ciliates, but there are diverse hosts where the symbiont’s phylogenetic position is unknown.

```
PREFIX pp: <https://ppsdb.wikibase.cloud/entity/>
PREFIX ppt: <https://ppsdb.wikibase.cloud/prop/direct/>
PREFIX pps: <https://ppsdb.wikibase.cloud/prop/>
PREFIX ppss: <https://ppsdb.wikibase.cloud/prop/statement/>
PREFIX ppsq: <https://ppsdb.wikibase.cloud/prop/qualifier/>
PREFIX ppsr: <https://ppsdb.wikibase.cloud/prop/reference/>
```

```
SELECT DISTINCT ?host ?hostLabel ?symbiont ?symbiontLabel ?parentClass ?parentClassLabel
?localizationLabel

WITH {

  SELECT DISTINCT ?host ?hostLabel ?symbiont ?symbiontLabel ?wditem ?localizationLabel WHERE {

  ?host pps:P19 ?interaction.

  ?interaction ppss:P19 ?symbiont.

  # Get parent taxon of the symbiont, and the item in Wikidata it is mapped to as ?wditem

  ?symbiont ppt:P29 ?parent.

  ?parent ppt:P2 ?wditem.

  ?interaction ppsq:P20 ?localization.

  # Qualifier: symbiont is localized in nucleus, or a subclass of or part of nucleus

  ?localization ppt:P24*/ppt:P47* pp:Q78

  # Labels for the retrieved items

  SERVICE wikibase:label {

    bd:serviceParam wikibase:language "[AUTO_LANGUAGE],en".

    ?host rdfs:label ?hostLabel.

    ?symbiont rdfs:label ?symbiontLabel.

    ?localization rdfs:label ?localizationLabel.

  }

  }

# Store results from the above SELECT block in an array %wd, to be passed to the subquery below

} AS %wd

WHERE {

  INCLUDE %wd

  # Set up a federated query: code in this SERVICE block will be sent to the Wikidata query server

  SERVICE <https://query.wikidata.org/sparql> {

    OPTIONAL {

      # Chained “parent taxon” statements to find higher taxa linked by P171 statements

      ?wditem wdt:P171* ?parentClass.

      # Parent taxon item of taxonomic rank “class”

      ?parentClass wdt:P105 wd:Q37517.

      SERVICE wikibase:label {

        bd:serviceParam wikibase:language "[AUTO_LANGUAGE],en".
```

```
      ?parentClass rdfs:label ?parentClassLabel.
    }
  }
}
} ORDER BY ?parentClassLabel ?hostLabel
```

Interactions characterized by FISH but not by sequencing

A number of symbionts have been described in publications that employed group-specific molecular probes that could identify them to e.g. class level, but which did not sequence a phylogenetic marker gene, so a more precise classification was not possible. These may be interesting to revisit with modern sequencing methods.

```
PREFIX pp: <https://ppsdb.wikibase.cloud/entity/>
PREFIX ppt: <https://ppsdb.wikibase.cloud/prop/direct/>
PREFIX pps: <https://ppsdb.wikibase.cloud/prop/>
PREFIX ppss: <https://ppsdb.wikibase.cloud/prop/statement/>
PREFIX ppsq: <https://ppsdb.wikibase.cloud/prop/qualifier/>

SELECT DISTINCT ?host ?hostLabel ?symb ?symbLabel WHERE {
  # Query for statements with qualifier P22 “method used to identify object”
  ?host pps:P19 ?s.
  ?s ppsq:P22 pp:Q10. # fluorescence in-situ hybridization
  ?s ppss:P19 ?symb.
  # NB: an interaction may be documented in multiple statements
  # Exclude statements where symbionts have been characterized by sequencing methods
  FILTER NOT EXISTS {
    ?host pps:P19 ?t.
    ?t ppss:P19 ?symb.
    ?t ppsq:P22 pp:Q13. # phylogenetic marker sequencing
  }
  FILTER NOT EXISTS {
    ?host pps:P19 ?u.
    ?u ppss:P19 ?symb.
    ?u ppsq:P22 pp:Q12. # metagenome sequencing
  }
  SERVICE wikibase:label { bd:serviceParam wikibase:language "[AUTO_LANGUAGE],en". }
```

```
}
```

Find symbioses described in publications by a specific author

We can query bibliographic metadata of the publications referenced in PPSDB such as authors and publication venues, via reference items mapped to Wikidata. Like the search by biological taxonomy, this is a federated SPARQL query. The search exploits the growing representation of publication and person data in Wikidata, which can be used for scientometric studies, such as investigating coauthorship networks.

```
PREFIX pp: <https://ppsdb.wikibase.cloud/entity/>

PREFIX ppt: <https://ppsdb.wikibase.cloud/prop/direct/>

PREFIX pps: <https://ppsdb.wikibase.cloud/prop/>

PREFIX ppss: <https://ppsdb.wikibase.cloud/prop/statement/>

PREFIX ppsq: <https://ppsdb.wikibase.cloud/prop/qualifier/>

PREFIX ppsr: <https://ppsdb.wikibase.cloud/prop/reference/>


SELECT DISTINCT ?host ?hostLabel ?symb ?symbLabel ?statedin ?wdref

WITH {

  SELECT DISTINCT ?host ?hostLabel ?symb ?symbLabel ?statedin ?wdref

  WHERE {

    ?host pps:P19 ?interaction.

    ?host rdfs:label ?hostLabel.

    ?interaction ppss:P19 ?symb.

    ?symb rdfs:label ?symbLabel.

    # Get the reference cited for this statement

    # There is a specific syntax for retrieving a reference in Wikibase

    ?interaction prov:wasDerivedFrom ?refnode.

    ?refnode ppsr:P23 ?statedin.

    # Reference items are mapped to a Wikidata item (URI)

    ?statedin ppt:P2 ?wdref.

  }

} AS %refs

WHERE {

  # Federated query with Wikidata

  INCLUDE %refs

  SERVICE <https://query.wikidata.org/sparql> {
```

```
    # Reference items in Wikidata with a specific author Q30513710
    ?wdref wdt:P50 wd:Q30513710.
  }
}
```
