# Supplementary material for: Database Release: PPSDB, a Linked Open Data Knowledge Base for Protist–Prokaryote Symbioses
Source: J Eukaryot Microbiol. 2025 Oct 10;72(6):e70049. doi: 10.1111/jeu.70049 (PMC12512223; doi:10.1111/jeu.70049)
Supplement: Supplementary file 2 — Table S1: Network metrics for symbionts reported from two or more host species, for the network of symbiont co‐occurrences in PPSDB. [file JEU-72-e70049-s002.docx]

**TABLE S1.** Network metrics for symbionts reported from two or more host species, for the network of symbiont co-occurrences in PPSDB.

| **PPSDB Qid** | **Name** | **PageRank** | **Local clustering coefficient** | **Node degree** | **No. host spp.** |
| --- | --- | --- | --- | --- | --- |
| Q558 | *Polynucleobacter necessarius* | 0.013162 | 0.252632 | 20 | 8 |
| Q141 | *Candidatus* Megaira polyxenophila | 0.016066 | 0.236467 | 27 | 8 |
| Q310 | *Candidatus* Armantifilum devescovinae | 0.017276 | 0.000000 | 7 | 7 |
| Q564 | *Candidatus* Protistobacter heckmanni | 0.009176 | 0.419048 | 15 | 4 |
| Q1508 | *Candidatus* Megaira venefica | 0.009651 | 0.285714 | 14 | 4 |
| Q1512 | *Caedimonas varicaedens* | 0.008460 | 0.466667 | 15 | 4 |
| Q1549 | *Preeria caryophila* | 0.007271 | 0.589744 | 13 | 3 |
| Q2060 | *Candidatus* Finniella dimorpha | 0.005565 | 0.777778 | 9 | 3 |
| Q332 | *Candidatus* Endomicrobium trichonymphae | 0.007391 | 0.400000 | 5 | 2 |
| Q194 | *Parachlamydia acanthamoebae* | 0.006720 | 0.000000 | 2 | 2 |
| Q1769 | *Pseudolyticum multiflagellatum* | 0.006808 | 0.606061 | 12 | 2 |
| Q979 | *Candidatus* Bandiella numerosa | 0.003449 | 1.000000 | 5 | 2 |
| Q579 | *Candidatus* Trichorickettsia mobilis subsp. extranuclearis | 0.004770 | 0.523810 | 7 | 2 |
